# Antiviral Activity of Glycyrrhizin against Hepatitis C Virus *In Vitro*

**DOI:** 10.1371/journal.pone.0068992

**Published:** 2013-07-18

**Authors:** Yoshihiro Matsumoto, Tomokazu Matsuura, Haruyo Aoyagi, Mami Matsuda, Su Su Hmwe, Tomoko Date, Noriyuki Watanabe, Koichi Watashi, Ryosuke Suzuki, Shizuko Ichinose, Kenjiro Wake, Tetsuro Suzuki, Tatsuo Miyamura, Takaji Wakita, Hideki Aizaki

**Affiliations:** 1 Department of Virology II, National Institute of Infectious Diseases, Tokyo, Japan; 2 Division of Gastroenterology and Hepatology, Department of Internal Medicine, Jikei University School of Medicine, Tokyo, Japan; 3 Department of Laboratory Medicine, the Jikei University School of Medicine, Tokyo, Japan; 4 Research Center for Medical and Dental Sciences, Tokyo Medical and Dental University, Tokyo, Japan; 5 Liver Research Unit, Minophagen Pharmaceutical Co., Ltd., Tokyo, Japan; 6 Department of Infectious Diseases, Hamamatsu University School of Medicine, Hamamatsu, Japan; University of Washington, United States of America

## Abstract

Glycyrrhizin (GL) has been used in Japan to treat patients with chronic viral hepatitis, as an anti-inflammatory drug to reduce serum alanine aminotransferase levels. GL is also known to exhibit various biological activities, including anti-viral effects, but the anti-hepatitis C virus (HCV) effect of GL remains to be clarified. In this study, we demonstrated that GL treatment of HCV-infected Huh7 cells caused a reduction of infectious HCV production using cell culture-produced HCV (HCVcc). To determine the target step in the HCV lifecycle of GL, we used HCV pseudoparticles (HCVpp), replicon, and HCVcc systems. Significant suppressions of viral entry and replication steps were not observed. Interestingly, extracellular infectivity was decreased, and intracellular infectivity was increased. By immunofluorescence and electron microscopic analysis of GL treated cells, HCV core antigens and electron-dense particles had accumulated on endoplasmic reticulum attached to lipid droplet (LD), respectively, which is thought to act as platforms for HCV assembly. Furthermore, the amount of HCV core antigen in LD fraction increased. Taken together, these results suggest that GL inhibits release of infectious HCV particles. GL is known to have an inhibitory effect on phospholipase A2 (PLA2). We found that group 1B PLA2 (PLA2G1B) inhibitor also decreased HCV release, suggesting that suppression of virus release by GL treatment may be due to its inhibitory effect on PLA2G1B. Finally, we demonstrated that combination treatment with GL augmented IFN-induced reduction of virus in the HCVcc system. GL is identified as a novel anti-HCV agent that targets infectious virus particle release.

## Introduction

Hepatitis C virus (HCV) infection is a major public health problem since most cases cause chronic hepatitis, hepatic cirrhosis and hepatocellular carcinoma. Current treatment of chronic hepatitis C is based on the combination of pegylated interferon-alpha (IFN-α) and ribavirin. However, approximately 50% of treated patients infected with genotype 1 do not respond, or show only a partial or transient response, and therapy causes significant side effects [1]. In Japan, glycyrrhizin (GL) preparations (stronger neo-minophagen C (SNMC)) have been used for more than 20 years as a treatment for chronic hepatitis patients who do not respond to IFN therapy.

GL is the major component of licorice root extract, and is composed of glycyrrhetinic acid. GL has been shown to possess several beneficial pharmacological activities, including anti-inflammatory activity [2], anti-tumor activity [3], anti-allergic activities [4], and anti-viral activities [5]. Several mechanisms of the GL-induced anti-inflammatory effect are reported, such as inhibition of thrombin-induced platelet aggregation [6], inhibition of prostaglandin E2 production [7] and inhibition of phospholipase A2 (PLA2) [8].

Many anti-viral effects of GL have been reported previously, for example, against herpes simplex type 1 (HSV-1) [9], varicella-zoster virus (VZV) [10], hepatitis A (HAV) [11] and B virus (HBV) [12], human immunodeficiency virus (HIV) [13], severe acute respiratory syndrome (SARS) and coronavirus [14], Epstein–Barr virus (EBV) [15], human cytomegalovirus [16] and influenza virus [17]. GL has been considered as a potential treatment for patients with chronic hepatitis C, and long term administration of GL to patients is effective in suppressing serum alanine aminotransferase (ALT) levels and histological change [18]. However, a direct anti-viral effect of GL against HCV has never been reported.

In this study, we evaluated the anti-HCV effects of GL, and demonstrated that GL targeted the release step of infectious HCV particles from infected cells. We found that the suppression of virus release by GL may be derived from its inhibitory effect on group 1B PLA2 (PLA2G1B). These findings suggest possible novel roles for GL in the treatment of patients with chronic hepatitis C.

## Materials and Methods

### Cell culture and reagents

The human hepatoma cell line, Huh7, and its derivative cell line, Huh7.5.1, provided by Francis Chisari (Scripps Research Institute, La Jolla, CA), were maintained in Dulbecco’s modified Eagle’s medium (DMEM) containing 10% fetal bovine serum (FBS) [19]. Huh7 cells harboring the subgenomic replicon [20] [21] were maintained in complete DMEM supplemented with 0.5 mg/ml G418 (Geneticin, Life Technologies Japan Ltd., Tokyo, Japan). GL (20β-carboxyl-11-oxo-30-norolean-12-en-3β-yl-2-O-β-D-glucopyranuronosyl-β-D-glucopyranosiduronic acid) and IFN-α were kindly provided by the Minophagen Pharmaceutical Co., Ltd., (Tokyo, Japan) and MSD K.K., (Tokyo, Japan) respectively. Oleyloxyethyl phosphorylcholine (OPC) (Cayman Chemical Company, Ann Arbor, MI), sPLA2IIA Inhibitor I (MERCK, Darmstadt, Germany), anti-Actin (Santa Cruz Biotechnology, Santa Cruz, CA) and anti-Human CD81 (BD Pharmingen, San Jose, CA) antibodies were purchased. The solvents were distilled water (GL), ethanol (OPC), and DMSO (sPLA2IIA inhibitor).

### Quantification of HCV core antigen and cell viability

The production of cell culture-produced HCV (HCVcc) has been previously reported [22]. Purification of LD has been previously reported [23]. The concentration of HCV core antigen in filtered culture medium, in cell lysates and in LD fraction of infected cells was determined using the Lumipulse Ortho HCV antigen kit (Ortho Clinical Diagnostics, Tokyo, Japan). Cell viability was analyzed by using Cell Titer-Glo Luminescent Cell Viability Assay (Promega, Madison, WI) according to the manufacturers’ protocol.

Electroporation of HCV RNA lacking E1 and E2

In vitro synthesis of HCV RNA JFH1 lacking E1 and E2 (JFH1delE1E2), and electroporation were performed as described previously [22].

### HCV pseudoparticle (HCVpp) assay

HCVpp harboring E1 and E2 glycoproteins of the JFH-1 clone (genotype 2a) (HCVpp2a) and the TH clone (genotype 1b) (HCVpp1b) were produced as previously described [24]. Pseudotype virus with VSV G glycoprotein (VSVpp) were also generated [24]. Huh7 or Huh7.5.1 cells were seeded into 48-well plates, incubated overnight at 37°C, and then infected with the HCVpp in the presence of various concentration of GL. Several hours post-infection, medium was replaced with DMEM with 10% FBS, and the cells were harvested 48 hours later to determine intracellular luciferase activity (Luciferase Assay System, Promega).

### HCV subgenomic replicon assay

The assay for the genotype 1b and 2a subgenomic reporter replicon has been previously reported [20] [21]. After 72 hours of treatment with GL, the replicon-transfected cells were harvested for either measurement of luciferase activity (Promega) or HCV RNA titer, as described previously [25]. The replication efficiency of HCV in each preparation was calculated as the percentage of luciferase activity or HCV RNA titer compared with that of cells subjected to the control treatment.

### Extra- and intracellular infectivity

To determine extracellular HCV infectivity, naïve Huh7 cells were inoculated with cell culture supernatant medium containing HCVcc. After 3 hours of incubation, the medium was replaced with DMEM containing 10% FBS, and the cells were cultured for an additional 72 hours. The infectious HCV titer in the culture medium was determined by quantification using the Lumipulse Ortho HCV antigen kit or by immunostaining of the HCV core antigen. Using an immunoassay that also provided results indicative of HCV infectivity [26], we confirmed a good correlation between the levels of core antigen and infectious titers (data not shown). To estimate intracellular infectivity, cells in the culture plates filled with DMEM containing 10% FBS were subjected to four cycles of freezing and thawing, using dry ice and a 37°C water bath. Cells in the culture plates were centrifuged at 1,200 rpm for 5 min at 4°C to remove cell debris, and the supernatants were collected to evaluate infectivity as above.

### RNA interference

The siRNA targeted to PLA2G1B, 5’- GCUGGACAGCUGUAAAUUUTT-3’, and scramble negative control siRNA to PLA2G1B were purchased from Sigma (Tokyo, Japan). Cells in a 24-well plate were transfected with siRNA using HiPerFect transfection reagent (Qiagen, Tokyo, Japan) following the manufacturer’s instructions.

Quantification of triglyceride

Triglyceride (TG) was measured with a Triglyceride kit (Wako, Tokyo, Japan) according to the manufacturer’s instructions.

### Indirect immunofluorescence assay

The inoculated cells were fixed with methanol and immunostained with a mouse monoclonal anti-core antibody and a rabbit polyclonal anti-NS5A antibody [22], followed by an Alexa Fluor 555-conjugated anti-mouse secondary antibody (Life Technologies Japan Ltd.).

### Transmission electron microscopy (EM)

Cells were fixed with 1.5% glutaraldehyde in 1.0% cacodylate buffer, pH 7.4, for 5 min, and then post-fixed with 2% OsO_4_ in phosphate buffer, pH 7.4, for 1 hour. The cells were dehydrated in ethanol and embedded in Epon. Ultrathin sections were double stained and examined at an accelerating voltage of 80 keV. Immuno-EM (IEM) were performed by using the labeled-(strept) avidin-biotin (LAB) kit according to the manufacturer’s instructions (Zymed laboratories, San Francisco, CA) as described previously [27].

### Statistical Analysis

Assays were performed at least four independent experiments. Data are expressed as the mean ± SD. Statistical analysis was performed using Student’s t test. 

## Results

### Anti-HCV effects of GL

**Figure 1:**
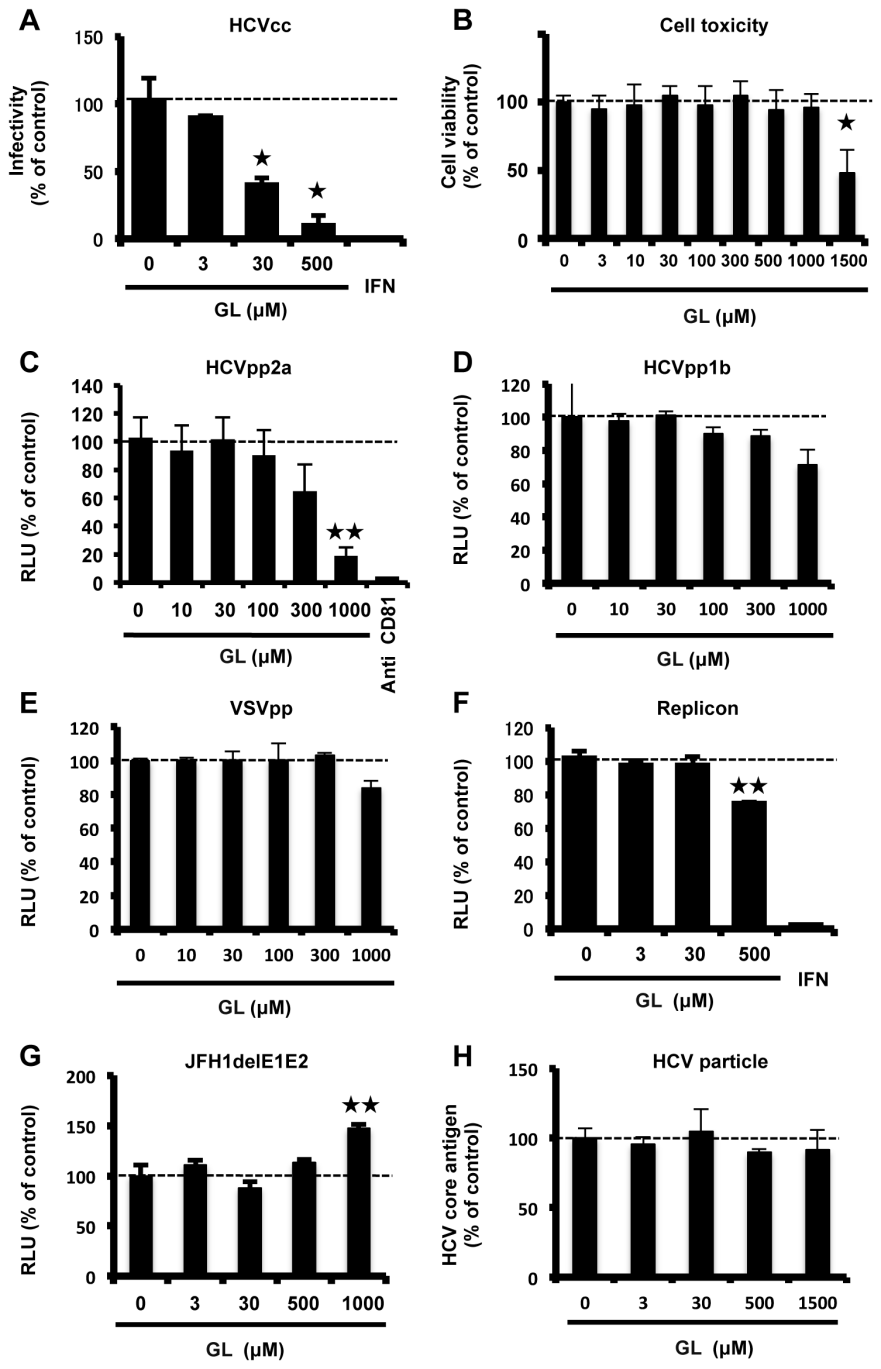
Anti-HCV effects of GL. (A) HCVcc-infected cells were treated with various concentrations of GL for 72 hours. Naïve Huh7 cells were inoculated with supernatant and cultured for 72 hours. Infectivity was determined by immunostaining. (B) Cell viability was assessed using Cell Titer-Glo Luminescent Cell Viability Assay. Huh7 cells were infected with HCVpp2a (C), HCVpp1b (D), and VSVpp (E) in various concentrations of GL for 24 hours, and then medium was replaced. Effects of GL on entry of HCVpp and VSVpp were determined by measuring the luciferase activity at 72 hours post-transfection. (F) Huh7 cells harboring the type-2a subgenomic replicon were treated with various concentrations of GL for 72 hours. Replication efficiency of the replicon was estimated by measuring the luciferase activity. (G) The effects of GL on HCV replication were tested by electroporation of HCV RNA lacking E1E2 (JFH1delE1E2). (H) HCV particles were treated with increasing concentrations (0 to 1500µM) of GL. The viral samples were then used to inoculate Huh7 cells with GL-containing medium. Several hours post-infection, medium was replaced with DMEM without GL. The levels of HCV core antigen of the medium were determined at 72 h postinfection (p.i.). IFN (300 IU/ml) was used as a positive control for reduced HCV replication. Anti-human CD81 antibody (10 µg/ml) was used as a positive control for reduced HCV entry to the cells. Results are expressed as the mean ± SD of the percent of the control from four independent experiments. *P < 0.05, **P < 0.005 versus control (0 µM treatment).

To assess the anti-HCV effects of GL, HCVcc-infected cells were treated with various concentrations of GL for 72 hours, and then the levels of HCV core antigen and infectivity of the medium were determined. HCV core antigen levels were reduced by 29% with 500 µM GL (Figure S1). As shown in Figure 1A, infectivity of supernatant following GL treatment at 3, 30, or 500 µM was reduced by 12, 62, or 71% of the control levels, respectively. The calculated 50% effective concentration (EC_50_) was 16.5 µM. There was no effect on cell viability after these treatments (Figure 1B). These results suggest that GL effectively inhibited the production of infectious HCV.

HCV propagates in hepatocytes throughout its lifecycle, including the stages of attachment, entry, uncoating, translation, genome replication, assembly, budding, and release. To investigate which step of the HCV lifecycle GL inhibited, we used the HCVpp system for evaluating attachment and entry, and the HCV replicon system for translation and genome replication. Treatment of HCVpp2a with GL resulted in a moderate reduction of luciferase activity in the cells infected with HCVpp, with an EC_50_ value of 728 µM (Figure 1C). On the other hand, there was no significant reduction of luciferase activity in the cells infected with HCVpp1b (Figure 1D) and VSVpp (Figure 1E). No cytotoxic effects of GL were observed (data not shown).

Huh7 cells harboring the type-2a subgenomic replicon were treated with various concentrations of GL for 72 hours. Relative luciferase activities of GL-treated cells were inhibited in a dose-dependent manner with an EC_50_ value of 738 µM (Figure 1F). A similar result was obtained by using the type-1b subgenomic replicon (data not shown). We also transfected HCV RNA lacking E1E2 (JFH1delE1E2) and monitored the effect of GL on HCV replication to avoid reinfection of Huh7 cells. There was no significant reduction of HCV RNA titers in the cells (Figure 1G). There was no significant cytotoxicity seen following these treatments (data not shown).

To investigate the effect of GL on entry, HCV particles were treated with increasing concentrations (0 to 1500µM) of GL. The viral samples were then used to inoculate Huh7 cells cultured in GL-containing medium. Several hours post-infection, medium was replaced with DMEM without GL. The levels of HCV core antigen in the medium were determined at 72 h postinfection (p.i.). There was no significant reduction of HCV production (Figure 1H). These results indicated that GL did not inhibited HCV entry and replication significantly.

### Effects of GL on infectious HCV particle release

**Figure 2:**
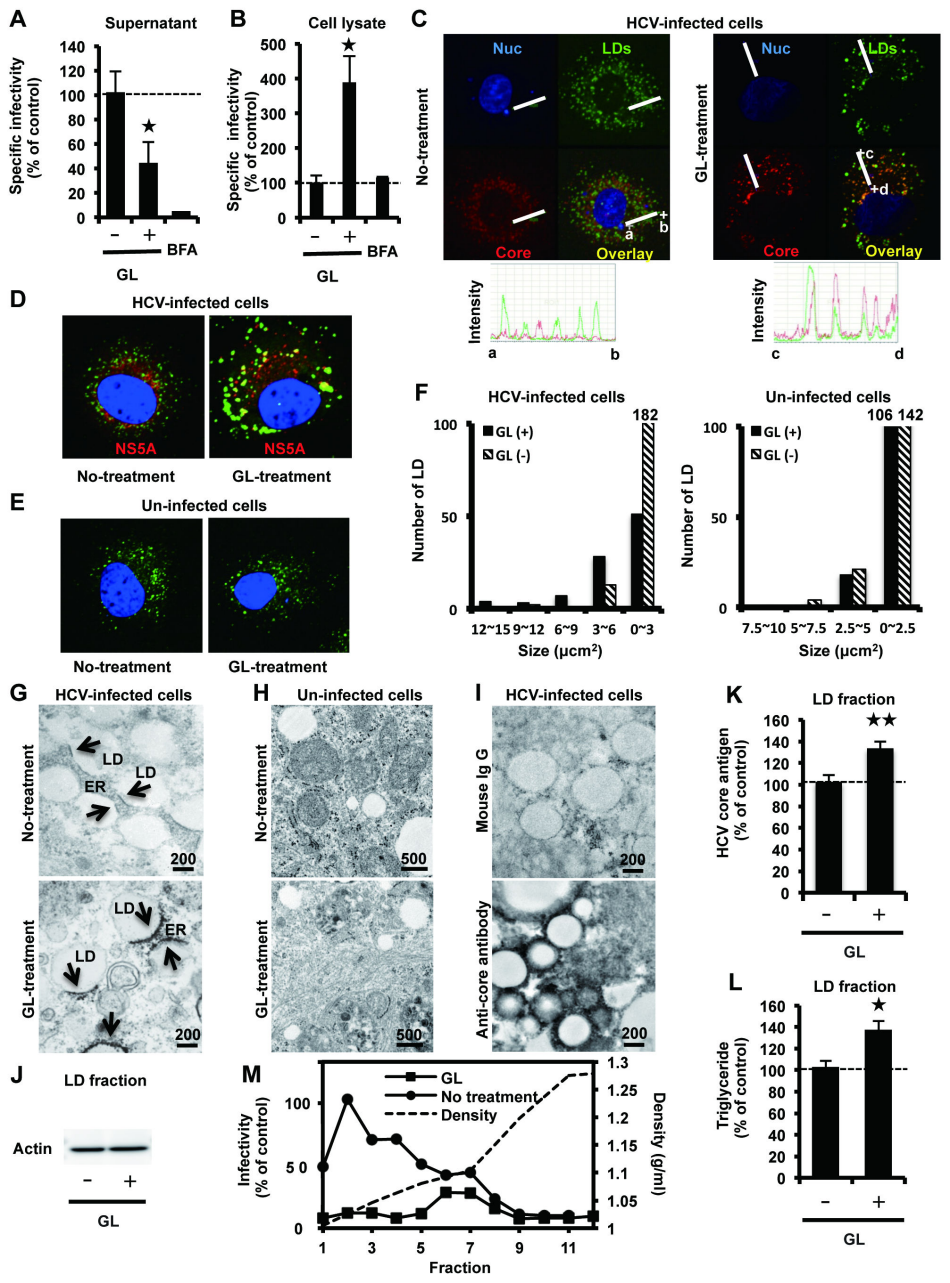
Effects of GL in release of infectious HCV particles. HCVcc-infected cells were treated with GL at a concentration of 500 µM for 72 hours. Untreated cells were used as controls. Extra- (A) and intracellular specific infectivity (B) were determined. Subcellular co-localization of HCV core (C) or NS5A (D) with LDs in HCVcc-infected cells with or without GL treatment. (E) Un-infected cells. LDs and nuclei were stained with BODYPI 493/503 (green) and DAPI (blue), respectively. (C) Points a and b, as well as c and d, define two line segments that each cross several structures. Intensity profiles along the line segments shown on the bottom of the images. (F) The size of LDs in un-ifected cells (right panel) and HCV-infected cells (left panel) were quantified. Transmission EM of LDs in infected cells (G) and un-infected cells (H) treated with GL at 500 µM. Arrows indicate electron-dense signals (G upper panel) and particles (G lower panel). (I) IEM using the LAB method of LDs in infected cells treated with GL at 500 µM. Mouse IgG (upper panel) or anti-core monoclonal antibody (lower panel) was used for primary antibody. (J) Immunoblotting with anti-actin antibody in the LD fraction. Quantification of HCV core antigen (K) and TG (L) in the LD fraction. The LD fraction was collected from cell lysates. The ratio of HCV core antigen level in the LD fraction to that in total cell lysate was determined. (M) HCVcc-infected cells were treated with GL at 500 µM for 72 hours. Untreated cells were used as controls. Supernatant was ultracentrifuged through a 10-60% sucrose gradient and the infectivity of each fraction was determined. Infectivity of fraction 2 of un-treated cells was assigned the arbitrary value of 100%. The density of each fraction was measured by refractive index measurement. Brefeldin A (1 µM for 24 hours) was used as a positive control for reduced HCV release. Results are expressed as the mean ± SD of the percent of the control from four independent experiments. *P < 0.05, **P < 0.005 versus control (0 µM treatment). Scale bars, 200 and 500 nm.

To further assess whether GL treatment affects other steps of the viral lifecycle, we analyzed infectious HCV particle assembly and release following GL treatment. Supernatant or crude cell lysates of HCVcc-infected cells treated with GL were used to inoculate naïve Huh7 cells to determine extra- and intracellular specific infectivity, respectively. Specific infectivity was determined as the ratio of infectious virus titer to HCV core antigen level, as described previously [28]. As shown in Figure 2A, the extracellular specific infectivity titer was inhibited by 57% by GL at a concentration of 500 µM, on the other hand, the intracellular specific infectivity titer was increased 3.8-fold over that of controls at the same concentration of GL (Figure 2B). There was no significant cytotoxicity following these treatments (data not shown).

It has been previously reported that virus assembly takes place around lipid droplets (LDs) [29]. By immunofluorescence staining, we examined the subcellular co-localization of HCV core (Figure 2C) or NS5A (Figure 2D) with LDs in HCVcc-infected cells with or without GL treatment. Un-infected cells were shown in Figure 2E. We observed HCV proteins colocalized with LDs (Figure 2C and 2D). Intensity profiles along the line segments, shown on the bottom of the images, demonstrated that core proteins were tightly colocalized with LD in the HCVcc-infected cells treated with GL, when compared with untreated cells (Figure 2C lower panel). We quantified the size of LDs in HCV-infected cells (Figure 2D) and un-infected cells (Figure 2E) with GL-treatment. We found that GL did not affect the size of LDs in un-infected cells (Figure 2F right panel). On the other hand, the size of LDs increased in HCV-infected cells with GL-treatment (Figure 2F left panel).

HCVcc-infected cells (Figure 2G) and un-infected cells (Figure 2H), treated with GL, were prepared for EM analysis. In the cytoplasm of HCV-infected cells, we observed increased numbers of LDs in close proximity to endoplasmic reticulum (ER) and the electron-dense signals on ER attached to LD (Figure 2G upper panel), which are thought to act as platforms for the assembly of viral components [29]. Interestingly, in the cytoplasm of HCV-infected cells after treatment with GL, accumulated electron-dense particles were observed on ER attached to LD (Figure 2G lower panel). IEM experiments showed that anti-core antibody stained the membrane around LDs (Figure 2I lower panel). In naïve Huh7 cells, the close association of LDs with ER was rarely observed (Figure 2H).

To confirm the accumulation of core antigen around LD, we purified the LD [23], and quantified HCV core antigen and TG in the LD fraction, followed by immunoblotting with anti-actin antibody (Figure 2J). Analysis of the levels of HCV core antigen and TG in the LD fraction of the total cell lysate showed that the amount in GL-treated cells was increased by 31% and 35% compared with controls, respectively (Figure 2K and 2L). Taken together, these results suggested that GL inhibits release, but not assembly and budding, of infectious HCV particles in cells.

To characterize the infectivity of HCV particles released from HCVcc-infected cells treated with GL, supernatant from cell cultures treated or not treated with GL was subjected to continuous 10-60% (w/v) sucrose density gradient centrifugation, and the infectivity titer of each fraction was measured. A reduction in infectivity by GL-treatment was observed in fractions 1-7 (Figure 2M). These results suggest that GL may decrease the amount of HCV infectious particles in the supernatant.

### Role of PLA2 in HCV lifecycle

**Figure 3:**
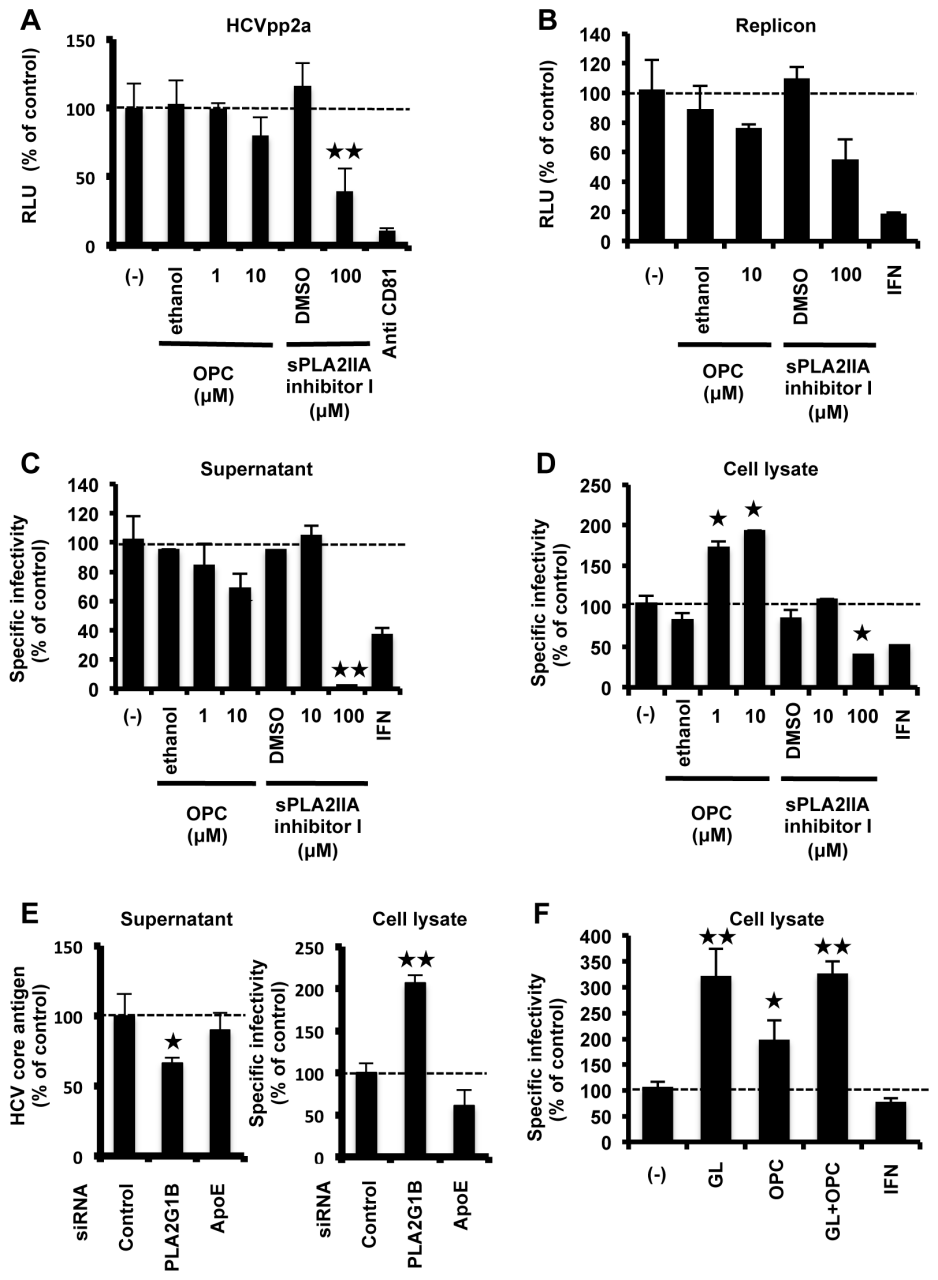
A role of PLA2 in HCV lifecycle. (A) Huh7 cells were infected with HCVpp in the presence and absence of OPC or sPLA2IIA inhibitor for 2 hours, then medium was replaced. Effects of PLA2 inhibitor on the entry of HCVpp were determined by measuring the luciferase activity at 72 hours post-infection. Anti-human CD81 antibody (10 µg/ml) was used as a positive control for reducing HCV entry to the cells. (B) Huh7 cells harboring the type-2a subgenomic replicon were treated with OPC or sPLA2IIA inhibitor for 72 hours. Replication efficiency of the replicon was estimated by measuring HCV RNA titer. HCVcc-infected cells were treated with PLA2 inhibitor for 72 hours. Specific infectivity of the supernatant (C) and cell lysate (D) were evaluated by quantifying the HCV core antigen in cells at 72 hours post-infection. (E) Effects of siRNA against PLA2G1B on core level in the medium (left panel) and specific infectivity in HCV-infected cells (right panel). ApoE siRNA was used as a positive control for reduced HCV infectivity. (F) HCVcc-infected cells were treated with GL (500 µM) with or without OPC (10 µM), and intracellular specific infectivity was measured. IFN (10 IU/ml) was used as a positive control. Results are expressed as the mean ± SD of the percent of the control from four independent experiments. *P < 0.05, **P < 0.005 versus control (0 µM treatment).

GL is known to have an inhibitory effect on PLA2 [8]. PLA2 is classified into several groups and their biological functions are not the same. It is unknown which group of PLA2 is targeted by GL. We analyzed the effect of GL on PLA2G1B and PLA2G2A, which were major groups of PLA2 family. To confirm the effects of GL on expression of PLA2G1B, cells, transfected with an expression plasmid for PLA2G1B, were treated with GL and OPC, which is a specific inhibitor for PLA2G1B. Treatment with GL effectively decreased the cellular level of PLA2G1B (Figure S2). To verify whether PLA2 has a role in viral entry and replication, we tested the effect of PLA2 inhibitors on HCVpp infection and the replicon system, respectively. OPC has no significant effect on virus entry and replication (Figure 3A and 3B). On the other hand, sPLA2IIA inhibitor I, which is a specific inhibitor for PLA2G2A, inhibited both HCVpp entry (Figure 3A) and subgenomic replicon replication (Figure 3B). There was no significant cytotoxicity seen after these treatments (data not shown).

To evaluate the effects of PLA2 inhibitors on HCVcc infectivity, infected cells were treated with PLA2 inhibitors and extra- and intracellular specific infectivity were measured (Figure 3C and 3D). OPC slightly decreased specific infectivity of virus in the supernatant and significantly increased specific infectivity of virus in the cell lysate. On the other hand, sPLA2IIA inhibitor I significantly decreased the specific infectivity of virus in both the supernatant and cell lysate. To confirm the importance of PLA2G1B in HCV release, we silenced PLA2G1B with its specific siRNA and monitored its effect on HCV release. PLA2G1B siRNA decreased the cellular level of PLA2G1B (Figure S3). Suppression of PL2G1B reduced core protein level in the medium (Figure 3E left panel) and increased specific infectivity in the cells (Figure 3E right panel). We performed GL treatment with or without OPC and showed that GL and OPC had no additive effect when applied together (Figure 3F). There was no significant cytotoxicity seen after these treatments (data not shown). Taken together, these results suggest that the suppression of virus release by GL may be derived from its inhibitory effect on PLA2G1B. These results also suggested that PLA2G1B has a role in virus release.

### Antiviral effects of IFN along with GL

**Figure 4:**
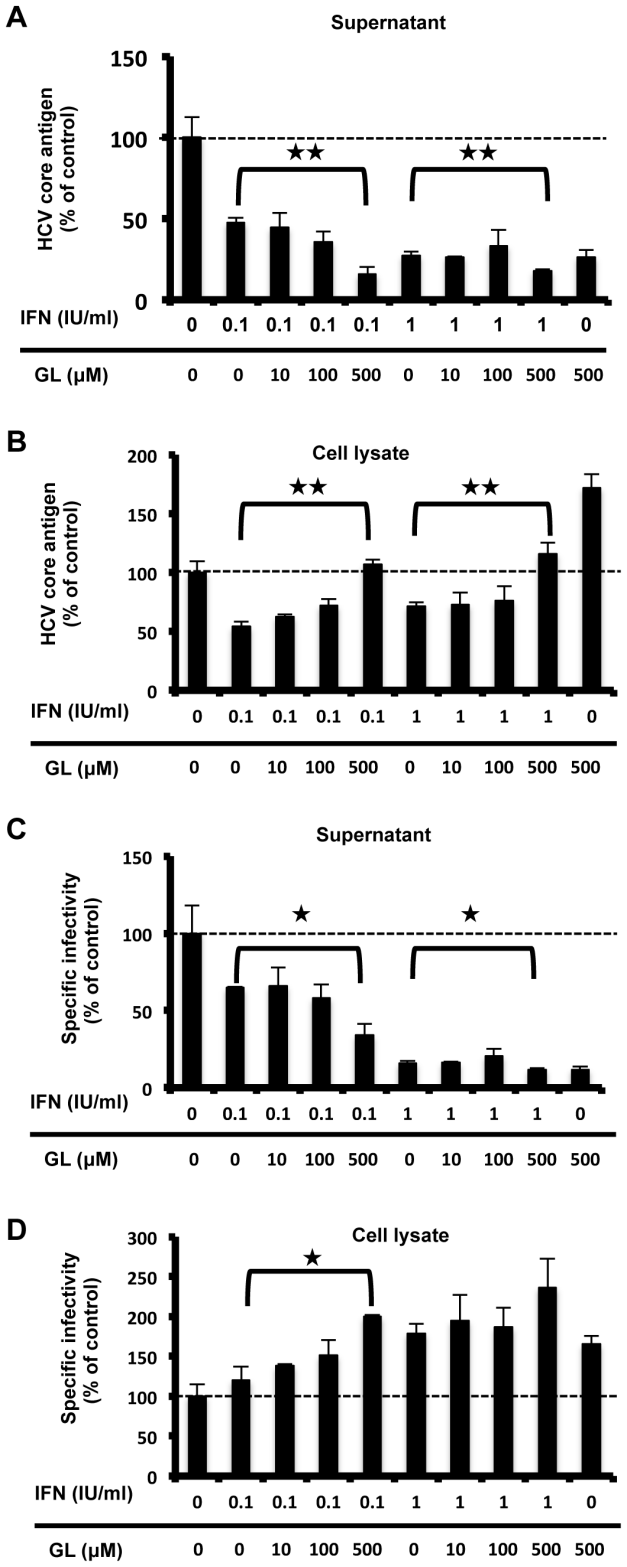
Anti-HCV effects IFN in combination with GL. HCVcc-infected cells were treated with IFN alone, or IFN with GL for 72 hours. HCV production was assessed by measuring the HCV core antigen in culture medium (A) and cell (B). Specific nfectivity in culture medium (C) and cell (D) were measured. Results are expressed as the mean ± SD of the percent of the control from four independent experiments. *P < 0.05, **P < 0.005 versus versus IFN mono-therapy.

We have demonstrated that the target causing the anti-HCV effect of GL differs from that of IFN. To analyze the antiviral effect of IFN combined with GL, HCVcc-infected cells were treated with 0.1 and 1.0 IU/ml of IFN in combination with various concentrations of GL. HCV core level in culture medium (Figure 4A) and in the cell (Figure 4B), specific infectivity in culture medium (Figure 4C) and in the cells (Figure 4D) were measured. Regardless to the IFN concentration, HCV core level and specific infectivity of the supernatant decreased in response to GL treatment in a dose dependent manner (Figure 4A and 4C). On the other hand, HCV core level and specific infectivity of the cell increased (Figure 4B and 4D), suggesting that GL inhibited HCV release. The results indicated that a combination therapy of IFN with GL could be an effective treatment for HCV.

### Effect of GL on IFN induction and secretion proteins

The IFN-inducing ability of GL has also been previously reported [30]. We evaluated IFN stimulated gene induction by GL, but no effects were observed (Figure S4). PLA2 is known to be associated with various intracellular trafficking events and secretion of very low-density lipoprotein (VLDL) [31]. HCV particles are known to be secreted using the host membrane trafficking system [32]. There is now increasing evidence that VLDL participates in HCV assembly and release [33]. Therefore, we analyzed the level of albumin, an abundantly secreted protein from hepatocytes, and apolipoprotein E (ApoE), a component of lipoproteins, in the culture supernatants of Huh7 cells and found that they were not influenced by GL treatment (Figure S5). 

## Discussion

Recently, Ashfaq et al. found the inhibitory effect of GL on HCV production in patient serum infected Huh7 cells [34]. Their cell culture system does not produce HCV efficiently. Thus, it does not permit analysis of the complete viral life cycle. In this study, we observed distinct suppression of HCV release by GL, using the HCVcc system (Figure 1A). Anti-viral effects of GL on early steps in the viral lifecycle have been reported previously, for example the inhibition of endocytosis of influenza A virus (IAV), the direct fusion of HIV-1 [35], the penetration of the plasma membrane of HAV [11] and EBV [15], the virus entry of SARS [14], and infection by pseudorabies virus [36]. GL effectively inhibits the replication of VZV [10], HSV-1 [9], EBV [15] and HIV [13]. This is the first report that GL can suppress virus release, however, the detailed mechanisms of these remain elusive. It has also been reported that GL had a membrane stabilizing effect [37] and a reduction of membrane fluidity [35], [38]. HCV uses cellular membrane structure in its lifecycle [39], [40]. Thus, it is conceivable that membrane alterations may play a negative role in the HCV lifecycle.

We found core protein accumulation on LDs in GL-treated cell (Figure 2C, 2I and 2K). This inverse correlation between the efficiency of virus production and core protein accumulation on LDs was also observed that colocalization of HCV protein with LDs was low in cases of the chimera Jc1, supporting up to 1,000-fold higher infectivity titers compared with JFH1 [41], [29]. In this study, we demonstrated that GL did not affect the size of LDs in un-infected cells (Figure 2F right panel). On the other hand, the size of LDs increased in HCV-infected cells with GL-treatment (Figure 2F left panel), probably because accumulated-HCV enhanced the formation of LDs [29].

We demonstrated the importance of PLA2G1B in HCV release by PLA2G1B inhibitor and siRNA against PLA2G1B (Figure 3). The overexpression of PLA2G1B did not have any effect on HCV release (data not shown), probably because enough PLA2G1B existed in the cells. This result is generally observed in other host factors that involved in HCV lifecycle. For example, overexpression of the human homologue of the 33-kDa vesicle-associated membrane protein-associated protein (hVAP-33), which has a critical role in the formation of HCV replication complex, did not increase HCV replication [42]. PLA2 family proteins have been known as lipid-signaling molecules, inducing inflammation [43]. On the basis of the nucleotide sequence, the superfamily of PLA2 enzymes consists of 15 groups, comprising 4 main types: cytosolic PLA2 (cPLA2), calcium-independent PLA2, platelet activating factor acetyl hydrolase/oxidized lipid lipoprotein associated PLA2, and the secretory PLA2 (sPLA2) including PLA2G1B, 2A, and 4A [44]. In this study, we showed that GL, PLA2G1B inhibitor, and PLA2G1B siRNA inhibited HCV release and that GL and OPC had no additive effect when applied together, suggesting that suppression of HCV release by GL may be derived from its inhibitory effect on PLA2G1B. The role of PLA2G1B in the HCV lifecycle has not been reported. In this study, we also demonstrated that PLA2G2A inhibitor decreased entry, replication, and assembly of infectious HCV particles in cells (Figures 3A, 3B, 3C, and 3D). The role of PLA2G2A in the HCV lifecycle has not been reported. PLA2G2A is known to affect the secretion of VLDL (30). Therefore, PLA2G2A may contribute to HCV assembly. In the case of PLA2G4A, Menzel et al. showed that inhibition of PLA2G4A produces aberrant HCV particles [45]. These observations suggest that PLA2 has a role in several steps of the HCV lifecycle.

In this study, we showed that the EC_50_ of GL treatment for intracellular infectivity was 16.5 µM (Figure 1A). It has been reported that the maximum peripheral concentration of GL in normal patients is 145 µM [46]. The placebo-controlled phase I/II trial revealed no significant effect on viral titer [47]. In vivo, accumulated HCV in GL treated cells may cause lysis and apoptosis of the cells, leading to the release of infectious particles in the circulation. This may be a major limitation to use GL mono-therapy against HCV infection in patients. On the other hand, combination treatment with GL augmented the IFN-induced reduction in HCV core antigen levels (Figure 4A).

Although a number of natural compounds with anti-HCV activities were identified in recent years (Silymarin, EGCG, Ladanein, Naringenin, Quercetin, Luteolin, Honokiol, 3-hydroxy caruilignan C, and other things) [48], many aspects concerning their mechanisms of action remain unknown. In this study, GL is identified as a novel anti-HCV agent that targets the release steps of infectious HCV particles. We found that the suppression of viral release by GL may be due to an inhibitory effect of PLA2G1B. These observations provide a basis for development of an improved IFN-based combination therapy against chronic hepatitis C.

## Supporting Information

Figure S1 Anti-HCV effect of GL. HCVcc-infected cells were treated with various concentrations of GL for 72 hours. HCV production was assessed by measuring the level of HCV core antigen in culture medium. Results are expressed as the mean ± SD of the percent of the control from four independent experiments. IFN (10 IU/ml) was used as a positive control. *P < 0.05, **P < 0.005 versus control (0 µM treatment).
(TIF)

Figure S2 Effect of GL on expression of PLA2G1B. A human PLA2G1B cDNA was inserted into the EcoRI site of pCAGGS, yielding pCAGPLA2G1B. Since there was no effective antibody to detect endogenous expression of PLA2G1B, 293T cells transfected with the pCAGPLA2G1B plasmid were treated with GL (500 µM) for 72 hours and lysed in lysis buffer, followed by immunoblotting with anti-PLA2G1B and anti-actin antibodies. OPC (10 µM) was used as a positive control to reduce PLA2G1B protein in the cells.
(TIF)

Figure S3 Effect of PLA2G1B siRNA on expression of PLA2G1B. HCVcc infected-Huh7 cells in a 24-well plate were transfected with siRNAs targeted to PLA2G1B and scramble negative control siRNA, followed by immunoblotting with anti-PLA2G1B and anti-actin antibodies.
(TIF)

Figure S4 Effect of GL on IFN induction. The pISRE-Luc vector contains the firefly luciferase reporter gene, downstream of the IFN-Stimulated Response Element (ISRE) cis-acting enhancer element. The pRL-TK vector contains the renilla luciferase reporter downstream of the herpes simplex virus thymidine kinase (HSV-TK promoter), and was used as an internal control. Huh7 cells transfected with the pISRE-Luc vector and the pRL-TK vector were treated with various concentrations of GL for 72 hours, and luciferase activities were measured using the Dual-Luciferase Reporter Assay System. IFN (300 U/ml) was used as a positive control. Results are expressed as the mean ± SD percent of the controls (treatment with IFN).
(TIF)

Figure S5 Effect of GL on secretion of lipoprotein and the host proteins. Huh7 cells were treated or untreated with GL at 500 µM for 72 hours. ApoE and albumin in the culture supernatants were measured by immunoblotting and ELISA, respectively. Results are expressed as the mean ± SD of the percent of the control from four independent experiments.
(TIF)
