# Supplementary figures and images for: Antiviral Activity of Glycyrrhizin against Hepatitis C Virus In Vitro
Source: PLoS One. 2013 Jul 18;8(7):e68992. doi: 10.1371/journal.pone.0068992 (PMC3715454; doi:10.1371/journal.pone.0068992)

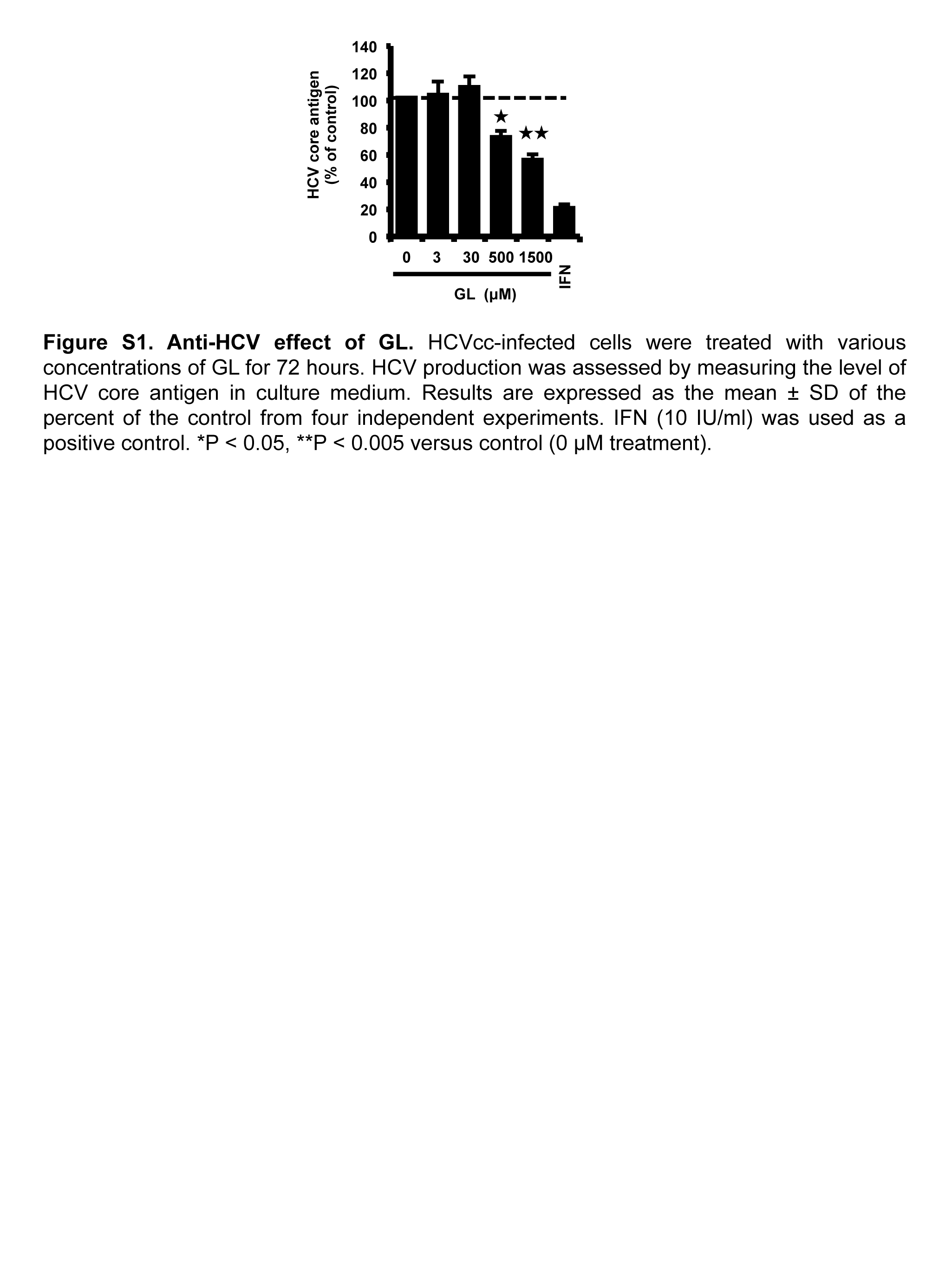

Supplement: Figure S1 — HCVcc-infected cells were treated with various concentrations of GL for 72 hours. HCV production was assessed by measuring the level of HCV core antigen in culture medium. Results are expressed as the mean ± SD of the percent of the control from four independent experiments. IFN (10 IU/ml) was used as a positive control. *P < 0.05, **P < 0.005 versus control (0 µM treatment). (TIF) [file pone.0068992.s001.tif]

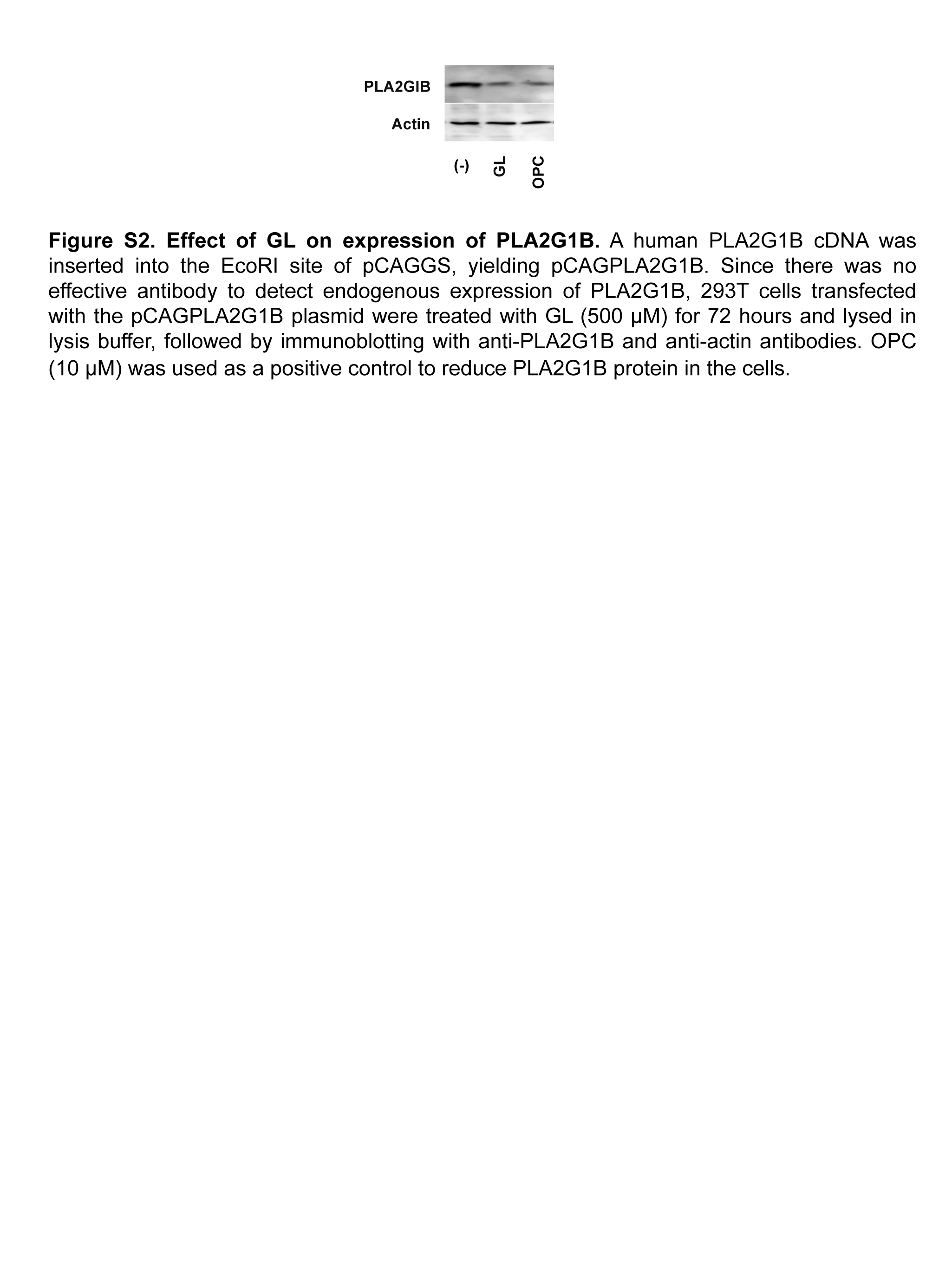

Supplement: Figure S2 — A human PLA2G1B cDNA was inserted into the EcoRI site of pCAGGS, yielding pCAGPLA2G1B. Since there was no effective antibody to detect endogenous expression of PLA2G1B, 293T cells transfected with the pCAGPLA2G1B plasmid were treated with GL (500 µM) for 72 hours and lysed in lysis buffer, followed by immunoblotting with anti-PLA2G1B and anti-actin antibodies. OPC (10 µM) was used as a positive control to reduce PLA2G1B protein in the cells. (TIF) [file pone.0068992.s002.tif]

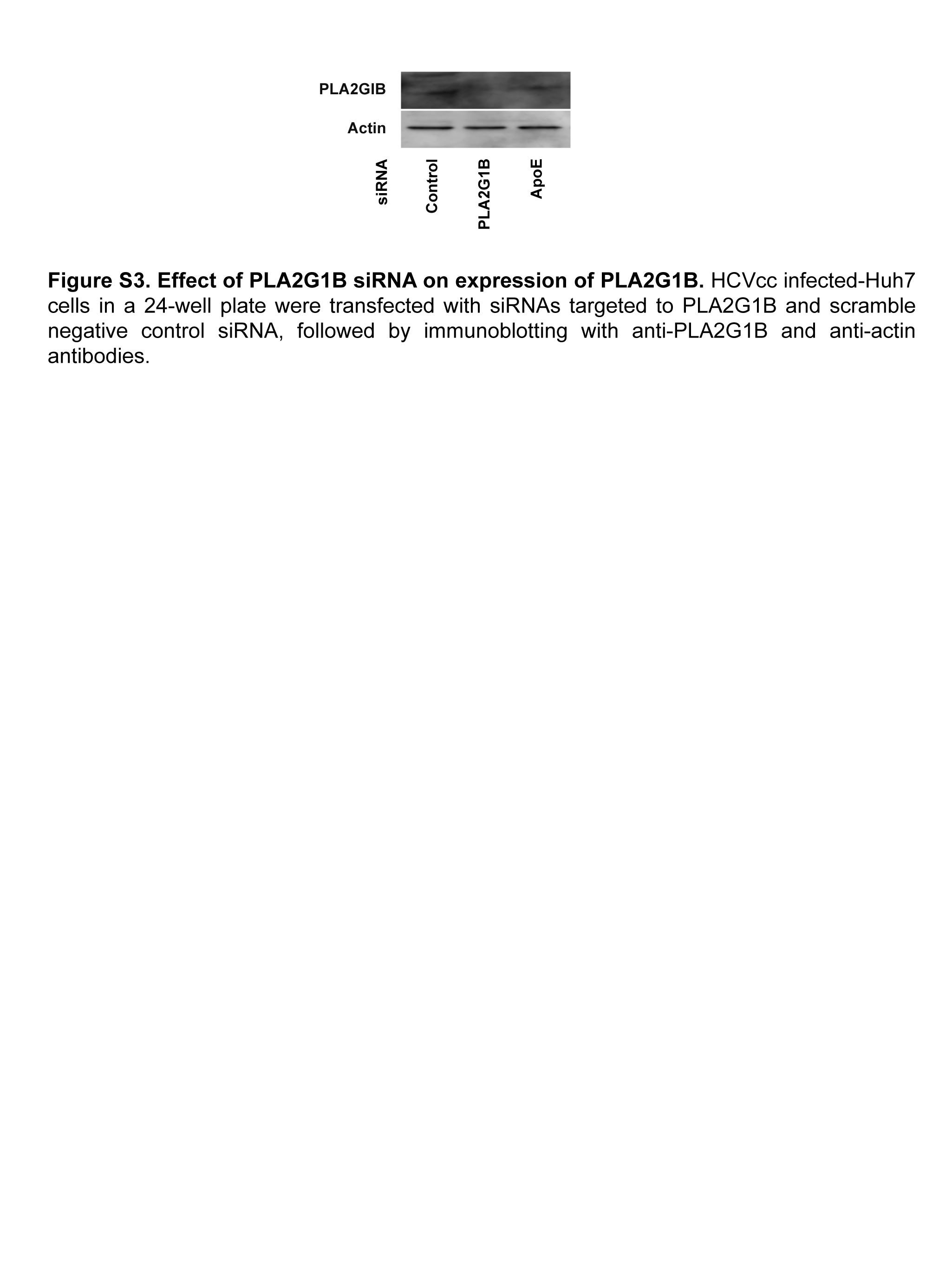

Supplement: Figure S3 — HCVcc infected-Huh7 cells in a 24-well plate were transfected with siRNAs targeted to PLA2G1B and scramble negative control siRNA, followed by immunoblotting with anti-PLA2G1B and anti-actin antibodies. (TIF) [file pone.0068992.s003.tif]

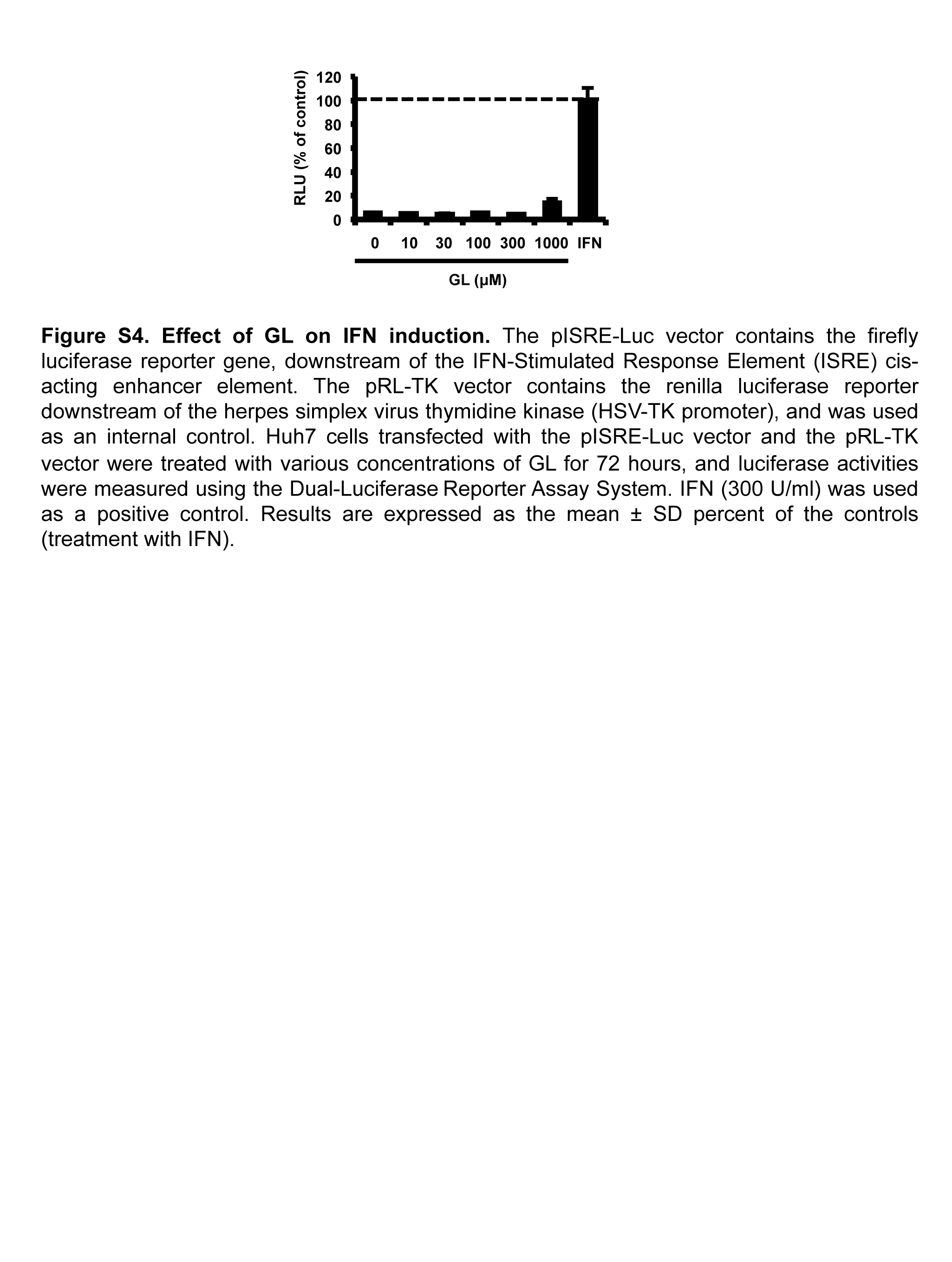

Supplement: Figure S4 — The pISRE-Luc vector contains the firefly luciferase reporter gene, downstream of the IFN-Stimulated Response Element (ISRE) cis-acting enhancer element. The pRL-TK vector contains the renilla luciferase reporter downstream of the herpes simplex virus thymidine kinase (HSV-TK promoter), and was used as an internal control. Huh7 cells transfected with the pISRE-Luc vector and the pRL-TK vector were treated with various concentrations of GL for 72 hours, and luciferase activities were measured using the Dual-Luciferase Reporter Assay System. IFN (300 U/ml) was used as a positive control. Results are expressed as the mean ± SD percent of the controls (treatment with IFN). (TIF) [file pone.0068992.s004.tif]

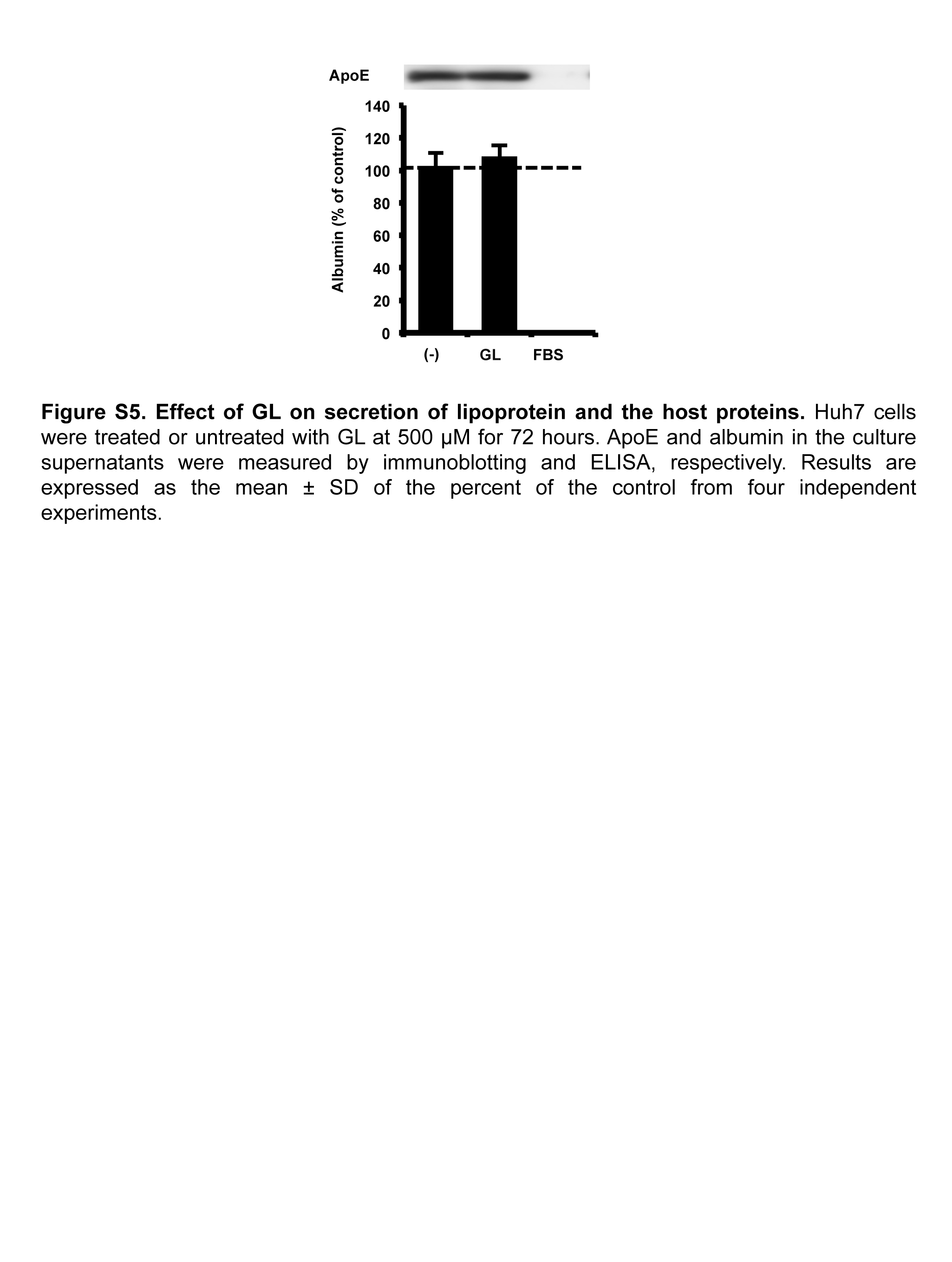

Supplement: Figure S5 — Huh7 cells were treated or untreated with GL at 500 µM for 72 hours. ApoE and albumin in the culture supernatants were measured by immunoblotting and ELISA, respectively. Results are expressed as the mean ± SD of the percent of the control from four independent experiments. (TIF) [file pone.0068992.s005.tif]
